# Automatic Localization of Seizure Onset Zone Based on Multi-Epileptogenic Biomarkers Analysis of Single-Contact from Interictal SEEG

**DOI:** 10.3390/bioengineering9120769

**Published:** 2022-12-05

**Authors:** Yiping Wang, Yanfeng Yang, Si Li, Zichen Su, Jinjie Guo, Penghu Wei, Jinguo Huang, Guixia Kang, Guoguang Zhao

**Affiliations:** 1 Key Laboratory of Universal Wireless Communications, Beijing University of Posts and Telecommunications, Ministry of Education, No. 10 Xitucheng Road, Haidian District, Beijing 100876, China; 2 Department of Neurosurgery, Xuan Wu Hospital, Capital Medical University, No. 45 Changchun Street, Xuanwu District, Beijing 100053, China

**Keywords:** stereo electroencephalogram (SEEG), preoperative evaluation, seizure onset zone (SOZ), deep learning, signal processing, pathological HFOs

## Abstract

Successful surgery on drug-resistant epilepsy patients (DRE) needs precise localization of the seizure onset zone (SOZ). Previous studies analyzing this issue still face limitations, such as inadequate analysis of features, low sensitivity and limited generality. Our study proposed an innovative and effective SOZ localization method based on multiple epileptogenic biomarkers (spike and HFOs), and analysis of single-contact (MEBM-SC) to address the above problems. We extracted contacts epileptic features from signal distributions and signal energy based on machine learning and end-to-end deep learning. Among them, a normalized pathological ripple rate was designed to reduce the disturbance of physiological ripple and enhance the performance of SOZ localization. Then, a feature selection algorithm based on Shapley value and hypothetical testing (ShapHT+) was used to limit interference from irrelevant features. Moreover, an attention mechanism and a focal loss algorithm were used on the classifier to learn significant features and overcome the unbalance of SOZ/nSOZ contacts. Finally, we provided an SOZ prediction and visualization on magnetic resonance imaging (MRI). Ten patients with DRE were selected to verify our method. The experiment performed cross-validation and revealed that MEBM-SC obtains higher sensitivity. Additionally, the spike has better sensitivity while HFOs have better specificity, and the combination of these biomarkers can achieve the best performance. The study confirmed that MEBM-SC can increase the sensitivity and accuracy of SOZ localization and help clinicians to perform a precise and reliable preoperative evaluation based on interictal SEEG.

## 1. Introduction

Although antiepileptic drugs can effectively control the seizures of most epilepsy patients, more than 30% cannot be cured, which is called drug-resistant epilepsy (DRE) [1]. For such DRE patients, the best treatment is to take surgical measures to remove or disconnect the epilepsy-causing brain region to reduce or prevent seizures [2,3,4,5]. Moreover, the brain region where clinical seizures are actually generated is called the seizure onset zone (SOZ) [6]. Thus, the key for successful surgery relies on accurate localization of the SOZ.

**Figure 1:**
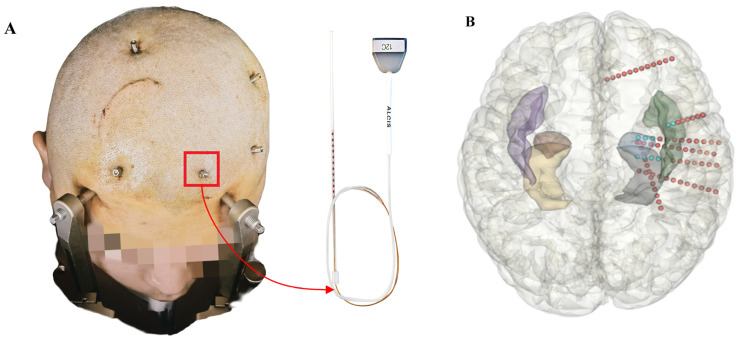
(**A**) is an intraoperative photo of electrodes after implantation, and the red frame contains a single electrode. Right side shows details of an SEEG electrode. The red points on the SEEG electrode are contacts. (**B**) depicts SEEG electrodes implanted in a whole 3D brain.

A stereo electroencephalogram (SEEG) is recorded by deep electrodes implanted in the deep brain [7], as shown in Figure 1; this differs from invasive electrocorticography (ECoG), and has been the gold standard for SOZ localization [8,9]. Specifically, clinicians place SEEG electrodes in suspicious areas which differ among patients, and bipolar conversion is required to minimize the correlation between two adjacent channels. Moreover, not only can SEEG record the electrical signal on the surface of the cerebral cortex but also the electrical data from the almond nucleus and the deep structure of the hippocampus. However, in clinical practice, clinicians usually prioritize checking the ictal period of SEEG, and its pathological information is quite limited. Patients may not even have seizures during hospitalization. In contrast, the interictal period of SEEG is easier to obtain and has much more information, ranging from a few days to even more than ten days. It is laborious, subjective, and time-consuming for clinicians to obtain further information by visually inspecting the long-range interictal SEEG [10]. Thus, an objective analysis method that can automatically estimate SOZ without the need for manual inspection of interictal SEEG has immense clinical significance [11,12,13,14,15]. Accordingly, this study aims to achieve objective and AI-based SOZ localization from interictal SEEG and to equip clinicians with trustworthy assistance for accurate preoperative evaluation.

Intelligent SOZ localization based on interictal SEEG and identification of epileptogenic contacts is typically accomplished in three steps: (1) The primary purpose is to detect potential epileptogenic markers in SEEG using algorithms such as signal filtering methods, machine learning, and deep learning; (2) Epileptogenic markers detected in the previous stage are used to extract contacts epileptogenic features; and (3) Feature selection is usually needed, and epileptogenic contacts classification is conducted based on machine learning algorithms.

In the first step of intelligent SOZ localization, epileptic markers’ detection plays a crucial role [14,16,17]. Research in recent years has suggested multiple promising biomarkers for SOZ localization, such as spikes [17], high-frequency oscillations (HFOs) (ripples (Rs), fast ripples (FRs), ripples co-occurring with FRs(R&FRs)) [8,18,19,20], and digital features of interictal epileptiform discharges [21,22,23]. Among these, for spikes detection, we demonstrated that deep learning can detect subtle changes in SEEG [24], and a more adaptive and highly interpretable SEEG-Net was then designed [25]. In addition, we have detected HFOs accurately from the filtered band-pass part and time-frequency image part, showing strong generalization ability and consistency with the gold standard [26,27]. Furthermore, for other important biomarkers, Akter et al. [22] detected the epileptic focus from interictal EEG using the information-theoretic features extracted from the high-frequency sub-bands. Klimes et al. [12] localized the SOZ in interictal SEEG by calculating multiple features of SOZ markers, such as oscillatory events and univariate spectral analysis. Therefore, improving the detection performance of epileptic biomarkers can lead to better SOZ localization and is of utmost importance. However, most studies can only determine if it is an epileptic signal, and cannot accurately trace the epileptogenic contacts and epileptogenic regions from the overall perspective of the individual patient. Thus, it was necessary to carry out a second step.

The key content of the second step is epileptogenic features extraction of contacts [13]. As using single biomarker is not enough to identify SOZ sensitively, Jacobs et al. [28] used HFOs to correct the seizure outcome of individual patients. Klimes et al. [12] proposed that the multi-feature approach is superior to the single-marker approach. It promoted the development of several markers’ combination for achieving a better identification of the SOZ. Thus, comprehensively represent the epileptogenic characteristics of contact based on multiple epileptogenic biomarkers is a future research trend. Furthermore, research shows that pathological and physiological HFOs largely overlap in their signal properties, and there is no reliable way to separate them. Physiological HFOs occur predominantly in the ripple range and can interfere with SOZ localization [17,29]. Based on this, Zweiphenning et al. [18] indicated that physiological ripple correction can enhance SOZ identification. Therefore, quantifying the pathological HFOs rate based on physiological ripple correction from the contact level to improve the performance of SOZ localization is an imperative issue for us to consider.

In the third step, effective feature selection is also challenging in SOZ classification [22,30]. In traditional clinical analysis, threshold methods are used [16,18,27]. Zweiphenning et al. [18] analysed various threshold methods, with the best method reaching 77.3% accuracy and 27% sensitivity. However, the threshold method still has problems, such as poor adaptability. In recent research combined with AI methods, Akter et al. [22] used sparse LDA to screen features and adopted machine learning classifiers such as SVM to classify SOZ contacts, achieved 52.70% sensitivity. The methods mentioned above still have shortcomings, such as lacking adaptability, and low sensitivity. In other fields, attention-based methods attracted the interest of researchers due to their advantages [24]. Therefore, designing a suitable feature selection method and classifier for SOZ identification is also crucial in our research.

Above all, we concentrated on tackling the following three issues to improve the sensitivity and comprehensive performance of SOZ localization based on the aforementioned constraints: (a) How to use end-to-end models to enhance the performance of epileptic biomarkers detection? (b) How to extract epileptic features comprehensively from the contacts level, and represent pathological ripple rate innovatively? (c) How to remove redundant features by designing a suitable feature selection method?

**Figure 2:**
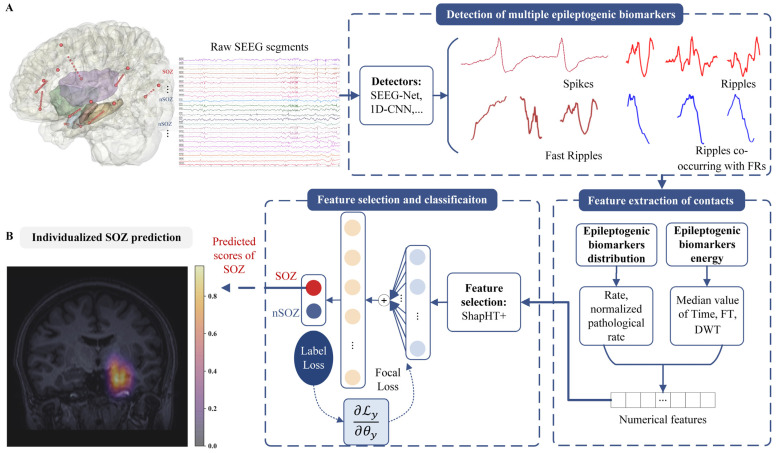
The pipeline of SOZ localization method: MEBM-SC: (**A**) the work scheme contains SEEG recording, multiple epileptogenic biomarkers detection, contact features’ extraction, feature selection and contact’s classification; and (**B**) interpolation of prediction scores onto patient’s individual MRI. Note: SOZ, seizure onset zone; nSOZ, non-seizure onset zone; FRs, fast ripples; FT, fourier transform; DWT, discrete wavelet transformation.

To overcome these issues, we present a highly sensitive and intelligent SOZ localization method based on multiple epileptogenic biomarkers analysis of single contact (MEBM-SC). Figure 2 depicts the whole MEBM-SC procedure. Specifically, (1) we calculated contacts features from two aspects, signal distribution, and signal energy. Among them, we propose a normalized pathological ripple rate of contacts based on ripple correcting [18] to minimize interference of physiological ripple and enhance performance of SOZ localization; (2) we introduced the Shapley value and hypothetical testing (ShapHT+) feature selection [31] algorithm to limit the interference from uncertain irrelevant features. (3) The attention mechanism (AM) and focal loss algorithms [32] were applied on a classifier to learn significant features and overcome the unbalance of SOZ/nSOZ contacts; and (4) To equip clinicians with dependable and interpretable assistance for precise presurgical evaluation, we provided SOZ prediction and visualization on magnetic resonance imaging (MRI).

The following is a summary of the main contributions of this study:

- We presented a high-sensitivity SOZ localization method based on multiple epileptogenic biomarkers analysis of single contact;
- We demonstrated that normalized pathological ripple rate with the regional +10% threshold can significantly improve the ability of SOZ identification;
- Machine learning and deep learning algorithms, such as SEEG-Net, AM, and Focal loss, were employed in each stage of the entire process. Cross validation was performed for stronger generality and generalization;
- Consistency was maintained with real clinical settings of preoperative DRE assessment. Individual SOZ prediction and visualization on MRI was provided to make reliable and explainable support.

## 2. Methods

### 2.1. MEBM-SC Overview

In this study, we proposed a novel and highly sensitive method for SOZ localization of DRE based on analyzing the interictal SEEG. MEBM-SC consisted of four parts: multiple epileptogenic biomarker detection; contact features extraction; contact features selection and classification; and individualized SOZ prediction and visualization. The implementation details are presented in Figure 2. The specific method is shown as follows.

### 2.2. Detection of Multiple Epileptogenic Biomarkers

Spike and HFOs (contain ripples (Rs) (80–250 Hz), fast ripples (FRs) (250–500 Hz) and ripples co-occurring with FR (R&FRs) (80–500 Hz)) are the main traditional and significant epileptogenic biomarkers for SOZs localization used in our study. Therefore, we use several verified models and algorithms to achieve high-sensitive detection of multiple epileptogenic biomarkers.

Specifically, our team applied three different pre-trained models and algorithms to detect spikes, both from traditional signal processing methods and deep learning models. Traditional signal processing methods include nonlinear feature extraction from the high-frequency bands [24], the specific features are presented in Table S1 in Supplementary Materials. For deep learning models, such as baseline model 1D-CNN [24] and the novel model SEEG-Net [25], we loaded the optimal parameters which had been pre-trained well to specific models. The model structure is presented in Supplementary Materials in Figures S1 and S2.

To detect HFOs, we improved the preliminary screening algorithm based on the HFOs detector [33]. Details are shown in Algorithm 1. The algorithm recognizes an incident with more than six oscillations in the specified time length, according to the amplitude of the Hilbert envelope of the band-pass filtering signal.
**Algorithm 1:** Preliminary Screening Algorithm**Require:** 1: *sigfull*: original signal; *sigfiltered*: bandpass filtered signal 2: *env*: Hilbert envelope of smoothed bandpass signal 3: *stdata*: *sigfull* 81–500 Hz Stockwell transform 4: $f_{s}$: Sampling frequency; x: any time; *Eol*: event of interest 5: *threshold*: ripple is 30, fastripple is 20**Ensure:**  7: $prob=\frac{{\left|stdata\right|}^{2}}{-\sum_{f\in \left[81,500\right]}\log\left[stdata\right]}$▷ Relative energy, the ratio of the energy occupied by a certain frequency at that moment  8: $S=-\sum_{f\in \left[81,500\right]}\log\left[prob\right]$▷ Signal energy entropy  9: **if** $\;\forall t\in \left[x,x+\frac{f_{s}}{100}\right]$, *sigfull*
≥ 0.9$S_{max}$
*& sigfiltered*
≤ 1010:   *baseline* ← *env(t)*; *baselineFiltered* ← *sigfiltered(t)*11:   *thr* ← *baseline* experience accumulation function 95% threshold12:   *thrFilt ← baselineFiltered* experience accumulation function 99% threshold13: **end if**14: **if** $\;\forall t\in \left[x,x+0.02f_{s}\right]$, *env(t)* ≥ 0.99thr
*&*
$env{\left(t\right)}_{max}<$*threshold*15:   *event* ← *env(t)*16:   *number* ← The number of times each *event* crossed *thrFilt*17:   **if** number≥ 618:     *Eol* ← *event*19:   **end if**20: **end if**21: **for** $\exists distance\left[Eol_{1},Eol_{2}\right]<10ms$ **do**22:   $Eol_{1}=Eol_{1}\cup Eol_{2}$23: **end for**

### 2.3. Multiple Features Extraction of Contacts

To extract much more comprehensive contacts features, we calculated multiple features based on the multi-biomarkers from two aspects: signal distribution and signal energy. A total of 126 features from multiple epileptogenic biomarkers were computed in this study, as shown in Table 1. In particular, we extracted normalized pathological ripple rates from contact level to reduce the impact of physiological ripple.

#### 2.3.1. Signal Distribution of Contact

##### Spike Rate

In this study, three different spike detectors were used: (1) a nonlinear feature extractor which has high accuracy and is explainable from high-frequency band; (2) 1D-CNN; and (3) SEEG-Net, which solved the problems of sample imbalance, domain shift across patients, and poor interpretability. This made it possible to detect pathological SEEG activity with high sensitivity. For a certain contact i of patient P, its spike rate $S_{rate}$ can be expressed as:

(1) $$S_{rate}=\frac{n_{S}{}_{j}}{T},j\in \left\{1,2,3\right\}$$

where $n_{S}$ means the count of spikes in contact i, j is the index of three models, and T is the time of calculation, it is thirty minutes in our study.

##### Normalized Pathological Ripples (Rs) Rate

In recent years, researchers found that the physiological ripples in HFOs have a greater impact on SOZ localization, and it is difficult to distinguish pathological and physiological ripples. Therefore, we no longer focus on distinguishing two types of signals. We extracted normalized pathological ripple rates from the contact level, which is of great significance in increasing localization sensitivity. The specific calculation is as follows:

For a certain contact i of patient P, its ripples rate $R_{rate}$ can be expressed as:

(2) $$R_{rate}=\frac{n_{R}^{i}}{T}$$

where $n_{R}^{i}\;$is the number of occurrences of ripples on contact i, and T is the total time of each segment was included in the study, the same as spike rate.

We noticed that normalization of the ripple substantially enhanced the capacity of ripples to identify SOZ zones, and the regional + 10% threshold (RT+10) demonstrated better performance, as in previous research [18]. Specifically, RT+10 was calculated by discarding contacts which Rs less than 10% of total number of Rs after regional correction. This was done to eliminate contacts whose HFO values were only marginally higher than physiological levels. Thus, the ripple rate after normalization $R_{normal\_r}$ can be expressed as follows:

(3) $$R_{normal\_r}=R_{rate}-R_{region}$$

(4) $$R_{normal\_r}=\{\begin{array}{cc} R_{normal\_r}, & if\;R_{normal\_r}\;in\;quantile\left(\overset{N_{c}}{\underset{i=1}{\sum }}n_{R}^{i},\left(0.1,1\right)\right)\\ 0, & if\;R_{normal\_r}\;in\;quantile\left(\overset{N_{c}}{\underset{i=1}{\sum }}n_{R}^{i},\left(0,0.1\right)\right)\\ 0, & if\;R_{normal\_r}<0\; \end{array}$$

where $R_{region}$ is a constant defined in previous studies [9] to represent different physiological ripple rates in different brain regions, the specific values of the brain regions involved in our dataset are shown in the Table 2. In addition, quantile(data,percentile) is used to set contacts which Rs less than 10% of the total number of Rs to 0. $N_{c}$ is the total number of contacts for the patient.

##### Fast Ripples (FRs) Rate

There are few physiological fast ripples, and physiological correction did not increase the effectiveness of FRs in locating SOZ. Thus, we calculated the feature directly. For a certain contact i of patient P, its fast ripple rate $FR_{rate}$ can be expressed as:

(5) $$FR_{rate}=\frac{n_{FR}^{i}}{T}$$

where $n_{FR}^{i}\;$is the number of occurrences of fast ripples on contact i.

##### Ripples Co-Occurring with FRs(R&FRs) Rate

The same as FRs, we calculate this feature based on the count of HFOs detection results. For a certain contact i of patient P, its R&FRs rate $R\&FR_{rate}$ can be expressed as:

(6) $$R\&FR_{rate}=\frac{n_{R\&FR}^{i}}{T}$$

where $n_{R\&FR}^{i}\;$ is the number of occurrences of R&FRs complex wave on contact i.

#### 2.3.2. Signal Energy of Contact

##### Spike Energy

We calculate the spike energy of contacts based on the handcrafted feature. Since the spike segment is 5s, we use the median value of the whole spike feature vectors in thirty minutes to represent the contact. For contact i of patient P, spike strength $S_{energy}\in {\mathbb{R}}^{n_{i}\times n_{f}}$ based on media value of DWT can be expressed as:

(7) Senergyi=M1median(fni,n∈[1,nSi ]),m∈[1,nf ]

where $n_{S}^{i}\;$is the number of occurrences of spike on contact I, $f_{n}^{i}$ is a feature vector, m is the number of feature types (e.g., Kraskov entropy, Renyi entropy).

##### Ripples Energy

Ripples energy of leads was extracted from three domains: (1) media value of Time; (2) median value of FT; and (3) median value of DWT. The calculation of the feature median is the same as Formula (7), and the specific feature extraction’s formula is shown in Table S2 of Supplementary Materials. Note that the length of HFOs is unfixed; we included temporal concentration in this part.

##### Fast Ripples Energy & R&FRs Complex Wave Energy

Since the length of fast ripples is shorter than ripples, we removed Kraskov entropy and Renyi entropy in the DWT domain. The other features in the study were precisely equated. The calculation of R&FRs complex wave energy is the same as Fast Ripples energy.

### 2.4. Feature Selection and Contact Classification

#### 2.4.1. Feature Selection Based on ShapHT+

To obtain more epileptogenic attributes and characteristics of the contacts, we extracted 126 characteristics from multiple aspects in 2.3. However, among them, there are some unrelated features and redundant information. Such features have no beneficial effect on contact classification performance and even reduce the operating efficiency of the model. Therefore, feature selection is necessary.

We use a feature selection strategy based on Shapley value and hypothetical testing (ShapHT+) [31]. First, we used the tree Shap algorithm [34] to calculate local and global importance and then design the adaptive threshold to evaluate the relevant features. Second, hypothetical tests were conducted for two essential indicators to exclude irrelevant features. The detailed algorithm steps are described below:

(a) We used the tree SHAP method to determine local importance. The algorithm is:

(8) $$T\left(X^{\left(n\right)}\right)=p_{0}+\overset{I}{\underset{i=1}{\sum }}p_{i}^{\left(n\right)}$$

(9) $$p_{i}^{\left(n\right)}=\underset{D\subseteq ℱ\backslash x_{i}}{\sum }\frac{\left|D\right|!\left(I-\left|D\right|-1\right)!}{I!}\left(g_{X^{\left(n\right)}}\left(X^{\left(n\right)}\cup x_{i}\right)-g_{X^{\left(n\right)}}\left(S\right)\right)$$

where D presents dataset and T(⋅) is a prediction model’s output value; $p_{0}$ is the expected output value, expressed as $p_{0}=E_{D}\left(T\left(X\right)\;\right)$; and $p_{i}^{\left(n\right)}$ denotes the contribution of feature $x_{i}$ in sample $X^{\left(n\right)}$ with the feature sets $ℱ=\left\{x_{i},i=1,\ldots ,I\right\}$; $g_{X}\left(D\right)$ represents the expectation of model T(⋅) conditioned on feature subset D in sample $X^{\left(n\right)}$. $\left|p_{i}^{\left(n\right)}\right|$, absolute value of contribution, is the local importance of feature $x_{i}$ in sample $X^{\left(n\right)}$, defined as $I_{i}^{\left(n\right)}$.

(b) Feature $x_{i}$’s global relevance is:

(10) $$GI_{i}=E\left(I_{i}\right)$$

where $I_{i}$ is the sequence of local importance $I_{i}=\left\{I_{i}^{\left(n\right)},n=1,\ldots ,N\right\}$; and $E\left(I_{i}\right)$ is the expected local importance in dataset D. $GI_{k}^{*}$ represents global importance of randomized feature $x_{k}^{*}$.

(c) We provide an adaptable threshold for assessing the relevance of features. As a result, the possibility that a feature is irrelevant is determined by computing proportion of features in sequence whose local relevance is lower than adaptive threshold. Hence, the final threshold is expressed as follows:

(11) $$Tre=c_{l}\cdot \max\left(GI_{k}^{*}\right),l=1,\ldots ,L$$

where L means maximum iterations and $c_{l}$ denotes the l-th iteration. $c_{l}$ can be adaptively adjusted until it reaches the maximum value.

(d) In addition, hypothesis tests were conducted to rule out irrelevant features. All the relevant characteristics were gathered as the initial features are sorted into relevant and irrelevant groups. The ideal feature subset had a good association with the label and little redundancy among features.

#### 2.4.2. Contact Classification Based on AM and Focal Loss

After feature selection, we used the significant features to complete the contact identification task. In real-world scenarios, the proportions of SOZ contacts and nSOZ contacts are particularly unbalanced and will impact the final localization outcome. Therefore, we adopted an attention mechanism (AM) based on shallow neural networks and modify the loss function to focus on learning the features of SOZ contacts. We resorted to focal loss, which was provided as a method for dealing with unbalanced data in dense object detection by [32].

Figure 2 shows the detailed structure of this part. To increase the effectiveness of DNN, which is specified by the following Formulas (12)–(14), AM is developed:

(12) $$u_{t}=\tanh\left(W_{w}e_{t}+b_{w}\right)$$

(13) $$h_{t}=\frac{exp\left(u_{t}^{T}u_{w}\right)}{\sum_{t}\exp\left(u_{t}^{T}u_{w}\right)}$$

(14) $$v_{t}=\underset{t}{\sum }h_{t}\cdot e_{t}$$

where $v_{t}$ is the attention layer’s output, while $W_{w}$, $u_{w}$ and $b_{w}$ represent trainable weights and bias. By multiplying $e_{t}$ and $h_{t}$, AM selects and extracts from $e_{t}$ temporal and spatial information that is most important to the detection tasks.

### 2.5. Individualized SOZ Prediction and Visualizaiton

As shown in Figure 2B, we map the predicted scores of the SOZ contacts to the patient’s personalized MRI, and the SOZ on MRI can be displayed more precise and more intuitive. The specific ideas are as follows:

- Considering the low correlation between brain boundary voxels and SOZ, this section set brain boundary voxels to the lowest quantified value;
- Considering the strong correlation between the SOZ surrounding voxels and SOZ contacts, we set SOZ contacts and their surrounding voxels to the predicted value corresponding to the contact;
- To further achieve whole-brain mapping, we used a 3D Gaussian kernel method to map all other voxels in the whole brain. The other voxels’ values are between the predicted values of the SOZ surrounding voxels and the quantified values of the brain boundary voxels;
- The predictive values of all voxels were calculated through the above interpolation method, ranging from 0 to 1. Then, we visualized the predictive values on the MRI. Thus, individualized SOZ prediction and visualization were obtained.

## 3. Experiments and Results

### 3.1. Patient Selection and Dataset Processing

We selected 10 consecutive patients receiving SEEG investigation followed by epilepsy surgery assessed at Xuanwu Hospital, China Department of Neurosurgery. The SEEG recordings are acquired with Nicolet at 2k/2048 Hz sampling rate, using depth DIXI electrodes. Table 3 provides comprehensive details on each patient.

Patients were required to meet the following criteria in this study:

(a) the patient has focal DRE;
(b) seizure free and outcome is Engel 1;
(c) a curative epilepsy surgery has been conducted;
(d) pathology is hippocampal sclerosis (HS);
(e) long-term SEEG monitoring, and post-operative structural MRI are obtainable;
(f) a minimum of six months of post-operative follow-up;
(g) at least one recorded seizure exhibits rhythmic activity.

We selected the interictal sleep state of ten patients with a single acquisition time of half-hour. The collection of sleep period data based on the patient’s video recording in which relative deep sleeping and turning over occurred are light. For dataset processing, the segmentation’s criteria and signal processing steps were the same as in the previous study [25]. Peri-implantation and postsurgical images were linearly matched to preimplantation MRI, and electrode placements are recorded. We utilized the same 17 regions as MNI Open iEEG Atlas to mark contacts region-specific HFO rates. Two neurophysiologists identified SOZ contacts based on the first unambiguous visual signal alterations after seizure onset.

### 3.2. Experimental Protocols

#### 3.2.1. Validation Protocols

**Figure 3:**
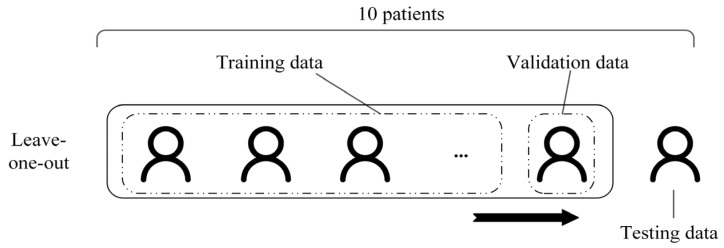
Leave-one-out cross-patient validation.

Leave-one-out cross-patient validation method was employed to achieve a robust evaluation and fine-tune parameters. The protocol is shown in Figure 3. The training data were constructed from 8 of 10 patients, and the remaining two patients constituted validation data and testing data, respectively. The final estimation was calculated by averaging the predictions from each split.

#### 3.2.2. Evaluation Metrics

Our study aimed to identify SOZ from the contact level. That is, this study aimed to identify SOZ/nSOZ contacts from all contacts planted in the brain, which becomes a binary classification task. Two neurophysiologists identified SOZ contacts based on the first unambiguous visual signal alterations after seizure onset. We used three overall metrics and two single-class metrics to evaluate the imbalanced classification performance for SOZ contact detection.

The specific formula is displayed as Formulas (15)–(19):

(15) $$\mathrm{Sensitivity}\left(\mathrm{SEN}\right)=\frac{TP}{\left(TP+FN\right)}$$

(16) $$\mathrm{Specificity}\left(SPE\right)=\frac{TN}{\left(TN+FP\right)}$$

(17) $$\mathrm{Accuracy}\left(\mathrm{ACC}\right)=\frac{TP+TN}{\left(TP+FP+TN+FN\right)}$$

(18) $$\mathrm{Positive}\;\mathrm{predictve}\;\mathrm{value}\left(\mathrm{PPV}\right)=\frac{TP}{\left(TP+FP\right)}$$

(19) $$\mathrm{Negative}\;\mathrm{predictive}\;\mathrm{value}\left(\mathrm{NPV}\right)=\frac{TN}{\left(TN+FN\right)}$$

where sensitivity (SEN) and specificity (SPE) represent the proportion of correctly identified SOZ and nSOZ contacts, respectively. Positive Predictive Value (PPV) measures the accuracy of all positive predictions. Negative Predictive Value (NPV) quantifies the proportion of accurate negative predictions when all negative predictions are included.

#### 3.2.3. Parameter Setting

The biomarkers deep learning detector, contacts feature selector and classifier may perform differently with different parameter values. For the parameters of multiple epileptogenic biomarkers detectors, the optimal values have been presented in the previous studies [24,25,26,27], respectively. All the settings for the contacts feature selector and deep learning classifier are shown in Table 4. All experiments were conducted with the PyTorch framework on DELL PowerEdge R740 rack servers with three-block NVIDIA GeForce RTX 2080 Ti.

### 3.3. Results for SOZ Localization Based on Cross Validation

**Figure 4:**
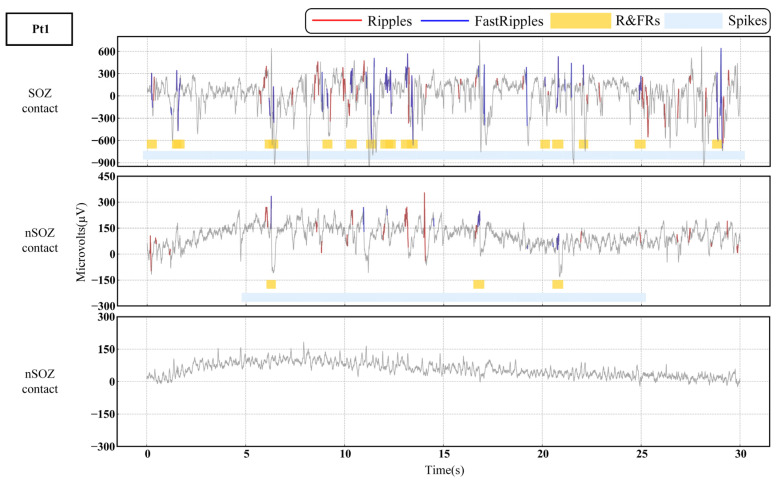
Raw interictal SEEG and multi epileptogenic biomarkers detection results plot in SOZ and nSOZ contacts for patient (Pt1).

**Figure 5:**
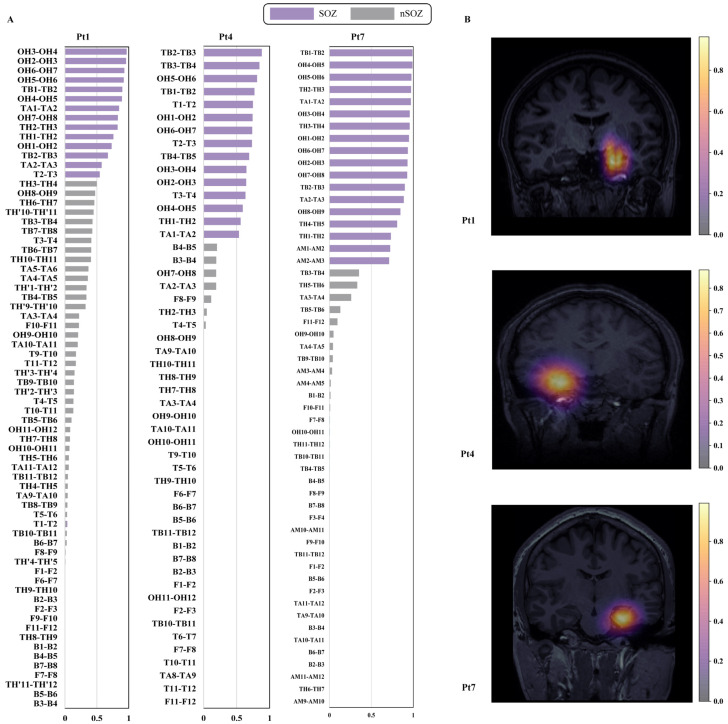
Bar-plot and MRI prediction probability for three patients: (**A**) bar-plot of contact prediction probability. X-axis and y-axis show prediction probability and contact name. Purple contacts are SOZ, while gray contacts are nSOZ; and (**B**) three patients’ predictive MRI.

We evaluated SOZ localization as a binary classification task and validated the proposed method MEBM-SC in the real-world, clinical DRE dataset, as shown in the Table 5. The method performed the best outcome for SOZ identification (89.27% sensitivity, 90.37% specificity, 90.87% accuracy, 81.38% PPV, 96.58% NPV). Specifically, first, end-to-end algorithms can enhance the performance of spike and HFOs detection. Figure 4 shows multi epileptogenic biomarkers detection results. Second, ripples correction can significantly improve the ability of single-contact analysis of SOZ identification. Third, the feature selection method and classifier are essential for our method. The prediction probability of different contacts is depicted in the bar plot and MRI in Figure 5.

### 3.4. Ablation Study

#### 3.4.1. Multi Biomarkers Features of Contacts

To investigate the contribution and improved performance of numerous biomarkers and normalized HFOs characteristics in our proposed MEBM-SC, we performed an ablation study. First, spike and HFOs features served as the foundational features, while the remaining features were progressively constructed as the whole feature set. Then, we integrated spike and HFOs. Moreover, pathological ripple rate was normalized. Finally, the normalized pathological ripple rate was replaced with a regional +10% threshold. In the process of conducting this ablation study, we maintained the feature selector and classifiers unchanged, the same as MEBM-SC. Particularly, the following describes the process:

- feature a (Multiple features of spike): we utilize the pathological signal distribution and energy of spike;
- feature b (Multiple features of HFOs): we employ the pathological signal distribution and energy of HFOs;
- feature c (Multiple features of spike and HFOs): we add the pathological signal distribution and energy of both biomarkers;
- feature d (Multiple features of spike and normalized pathological HFOs): add the normalization of ripples by the regional atlas threshold, based on feature c;
- feature e/MEBM-SC (Multiple features of spike and regional+10% based on normalized pathological HFOs): add the normalization of ripples by the regional + 10% threshold, based on feature c.

Table 6 demonstrates that the significant features of our method, particularly feature e, are effective for SOZ contact identification. This ablation study indicates that: (a) in general, the more comprehensive feature extraction, the higher the performance of SOZ contact recognition; (b) as illustrated above, the accuracy and sensitivity of feature c are higher than feature a and b, indicating that the combination of spike and HFOs is more effective. Conclusion is not clear yet as to which marker is more effective, in the previous study. The specificity of feature b is higher than all methods, indicating that the error rate of high-frequency oscillation is the lowest; and (c) using the regional +10% threshold demonstrates the highest performance. Both the sensitivity and accuracy of feature e are higher than other features, indicating the effectiveness of the ripple normalization, which is also consistent with the conclusions in Zweiphenning’s study [18].

#### 3.4.2. Feature Selection Methods

We discussed the effectiveness of the feature selection method and classifier with other methods, in this ablation study. Descriptions are as follows:

- selection a (XGBoost): exploit XGBoost as feature selection method and classifier;
- selection b (RFE-XGBoost): applie SVM-RFE as feature selection method and classifier;
- selection c (ShapHT++DNN): employ a shallow deep learning model, DNN as the feature selection method and classifier;
- selection d (ShapHT+self-AM+DNN): add self-attention machine based on selection c;
- selection e/MEBM-SC (ShapHT+AM+DNN): add attention machine based on selection c.

Table 7 shows the performance increase observed in selection e, showing that the proposed feature selection method and classifier are essential for our pipeline. As illustrated in selection c, shapHT+ significantly outperforms the sensitivity of selection a and b. The difference between selection d and e is the self-AM. Although both have the same level of sensitivity, selection d has lower specificity due to its poor fitting performance to negative samples. In conclusion, the ablation experiment demonstrated the effectiveness of ShapHT+ and AM.

## 4. Discussion

This study demonstrated that our proposed MEBM-SC can detect the difference between SOZ and non-SOZ contacts and achieve AI-based, high-sensitive SOZ localization (Table 8). Specifically, we confirm that: (a) Ripples correction with the regional +10% threshold can significantly improve the ability of SOZ identification; (b) Single-contact analysis of SOZ localization based on the spikes has better sensitivity, while analysis based on HFOs has better specificity, and biomarkers combination can achieve the best performance; and (c) SOZ contacts prediction and visualization on MRI can assist the epileptologist in making a prompt medical diagnosis; and (d) Cross-validation was used to verify the robustness and enhance the clinical availability of MEBM-SC.

### 4.1. Comparison with Other Methods

This study developed an SOZ localization method based on multi-epileptogenic biomarkers analysis of single contact. The summary of the mean outcomes of a 10-fold cross-patient evaluation can be found in Table 8. This table demonstrates that, MEBM-SC obtains the best performance on SEN (89.27%) and ACC (90.87%). The sensitivity is higher than the existing method, showing that our method predicts the best performance of SOZ contacts and could be more sensitive to discover abnormal contacts. In addition, the accuracy rate is higher, meaning that the comprehensive evaluation performance is better. The specificity is 90.37%, indicating that it can still maintain a lower false positive rate. Although these results are not directly comparable due to their different experiment settings, such as the number of patients, division of the dataset, and data preprocessing steps), the simple and broad comparison here can still suggest some empirical observations.

It is worth noting that, machine learning and deep learning algorithms are employed at each stage of the entire process. Firstly, for the detection of epileptogenic biomarkers, we used several algorithms to complete spike detection, such as 1D-CNN, the latest model SEEG-Net, and multi-band features, which were derived from our previous research. To precisely detect HFOs, we designed a morphological detection method based on the Hilbert envelope and achieve rapid detection of Rs, FRs, and R&FRs. Secondly, in the contacts feature extraction, we calculated the signal distribution features and signal energy features of multi-biomarkers in different contacts by signal processing technology. Thus, the epileptogenicity of contact is represented adequately. Thirdly, we employed the ShapHT+ algorithm, which is based on the Shapley value and adaptive relevance evaluation for feature selection. Each candidate feature assesses its relevance using the tree SHAP strategy. An adaptive threshold is applied to determine whether or not the feature is relevant to the a priori knowledge, and then binomial hypothesis testing was utilized to select all relevant characteristics. ShapHT+ outperform other algorithms in excluding interferences and selecting relevant features, as demonstrated in 3.4.2. Finally, for SOZ contacts identification, we introduced a shallow DNN with an attention mechanism and focal loss to improve DNN’s performance by focusing on the most discriminative input feature.

In addition, we verified the entire method by cross-validation. Such training and validation methods can avoid individual differences and large deviations in the clinical scenes. For example, some patients discharge more in the interictal period, and some discharge less. Moreover, the robustness is enhanced.

### 4.2. Significant Numerical Features of Epileptogenic Biomarkers

The critical part of SOZ localization is accurately representing the epileptogenicity of contact based on effective feature extraction. In this section, we analyzed the difference in significant features between SOZ and non-SOZ contacts.

The extraction of objective quantitative features of normalized pathological ripples rate from the contact level is an innovative point in this study. Normalization by the regional + 10% threshold substantially enhanced the ripples’ ability to identify the SOZ, exhibiting the best performance (3.4.1).

**Figure 6:**
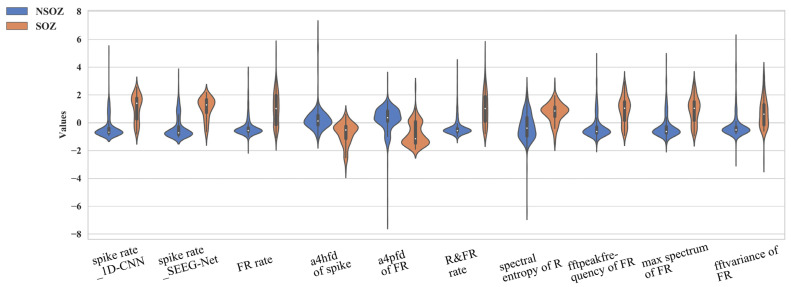
The ranking of the top ten most important features for patients.

We utilize ShapHT+ to evaluate the significance of features and to identify significant features. As illustrated in Figure 6, we sorted the 10 most vital features. Specifically, in the contact’s signal distribution group, the spike rate from 1D-CNN and SEEG-Net, Fast Ripple rate, and R&FRS rate are more significant, representing the occurrence rate of multi biomarkers. It is possible to observe the distinction between the two classes (SOZ/nSOZ). Besides, in the contact’s signal energy group, the most prominent features are a4Hfd of the spike, spectral entropy of ripples, a4Pfd/fftpeakfrequency/maxspectrum/fftvariance of FRs. Specifically, the HFD and PFD are implementations for calculating the FD in SEEG time series data. The fftpeakfrequency means the difference between the maximum and minimum of SEEG data after fast Fourier transform. The maxspectrum means the maximum absolute value of SEEG data after fast fourier transform. The fftVariance means the variance of SEEG data after fast fourier transform. Experiments indicated that these characteristics can differentiate SOZ contacts from non-SOZ contacts.

### 4.3. Extraction of Normalized Pathological HFOs Rate

Evaluating HFOs effectiveness is a challenge when considering physiological HFOs. Physiological HFOs mainly occur in ripple. In the interictal period, it is related to the cognitive process or is caused by tasks or stimuli. Pathological and physiological HFOs has overlapping in the signals’ nature, and there is no reliable way to separate them. The frequency of physiological waves has a significant difference in different brain regions. In addition, physiological FRs are rare.

Therefore, for physiological ripples, our study has quantified the correction of physiological HFOs, to obtain the pathological HFOs rate in the contact level, which can improve the predictive performance of the identification of SOZ. Specifically, the pathological HFOs rates of different areas are defined as the part exceeding each region’s standard physiological HFOs rate.

### 4.4. Visualization of Electrodes with MRI

**Figure 7:**
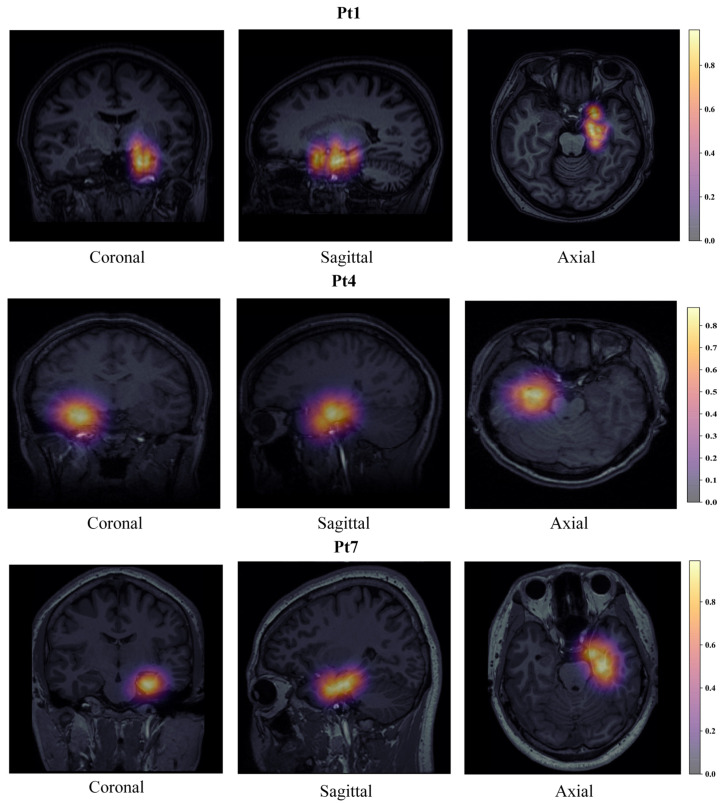
The SOZ prediction and visualization result with MRI from multiple axes.

In this section, we provide the SOZ prediction and visualization on the MRI, which can be seen in Figure 7. We make it possible to diagnose SOZ contacts more objectively and reliably. In addition to this, it can assist epileptologists with support in making quick medical judgments based on scores of contacts that have been mapped into the brain regions through the use of MRI.

### 4.5. Limitations and Future Work

Our proposed method in this study has potential, but it also has limitations: (1) From the perspective of computer-aided technology, the coupling between multiple modules did not perform very well. We will enhance the coupling between signal pattern detection and contact identification in future research, and realize an end-to-end identification method from raw SEEG to auxiliary diagnostic results (SOZ/nSOZ); (2) From the perspective of improving diagnostic performance, SOZ identification of single contact from interictal SEEG is of great significance. However, if brain network-based connectivity quantification can be taken into account, it would be helpful not only to have a significant performance improvement but also for the comprehension and application of intelligent-assisted SOZs localization; (3) From the perspective of comprehensive preoperative evaluation, it is also critical to locate SOZ of DRE based on the non-invasive images. In subsequent research, we will consider adding multi-modal data for analysis, such as (MRI, PET, etc.).

## 5. Conclusions

An AI-based and highly sensitive SOZ localization method, MEBM-SC, for diagnosing drug-resistant epilepsy utilizing interictal SEEG was proposed in this study. We focused on multi-epileptogenic biomarkers analysis of single-contact, such as spike and HFOs (Rs, FRs, and R&FRS). First, we extracted contacts features from two aspects, signal distribution and signal energy. Among these, we presented a normalized pathological ripple rate from the contact level. Second, we utilized the ablation study to demonstrate that integrating normalized pathological ripple rate, the combination of multi-biomarkers, and feature selection algorithms is available and suitable. Furthermore, cross-validation on clinical DRE databases indicated that MEBM-SC provides better results. Last, in order to supply clinicians with dependable and intelligible assistance for precise presurgical assessment, we provided the SOZ prediction and visualization on MRI, which can illustrate the possible SOZ contacts much easier and reliably.

## Figures and Tables

**Table 1:** Different features of contacts from two aspects: signal distribution and signal energy.

	Multiple Features	Signal Distribution	Signal Energy
Biomarkers			
**Spikes**		Rate/min (Feature based,1D-CNN, SEEG-Net)	Media value of DWT (Kraskov entropy, Renyi entropy, Permutation entropy, Sample entropy, Shannon entropy, Energy, SVD entropy, Petrosan Fractal Dimension (PFD), Katz Fractal Dimension (KFD), Higuchi Fractal Dimension (HFD)
**Hfos**	**Ripples**	Rate/min Normalized pathological ripples rate/min	Media value of Time (Energy, Time) Media value of FT (Maximum, Mean, Minimum, Peak frequency, Power, Power spectral density (Psd), Spectral entropy, Spectrum, Variance) Media value of DWT (Kraskov entropy, Renyi entropy, Permutation entropy, Sample entropy, Shannon entropy, Energy, SVD entropy, PFD, KFD, HFD)
	**Fast Ripples**	Rate/min	Same as ripples
	**Ripples co-occurring with FRs**	Rate/min	Media value of Time (Energy, Time) Media value of FT (Maximum, Mean, Minimum, Peak frequency, Power, Power spectral density (Psd), Spectral entropy, Spectrum, Variance) Media value of DWT (Permutation entropy, Sample entropy, Shannon entropy, Energy, SVD entropy, PFD, KFD, HFD)

**Table 2:** Different ripple rate in different region.

Index	Region	95th Percentile
2	Medial and basal temporal region	19.5
7	Superior, middle, and orbital frontal gyri and anterior part of inferior frontal gyrus	3.5
8	Middle and inferior temporal gyrus, temporal pole, and planum polare	2.7
9	Anterior and middle cingulate	2

**Table 3:** The clinical dataset’s patient characteristics.

Patient Id	Age	Sex	Number of SEEG Electrodes	Surgery Type	Outcome (Engel)	Follow-Up (Months)	Number of SOZ Contacts	Number of nSOZ Contacts
1	38	F	8	RF-TC	I	33	15	55
2	35	M	7	RF-TC	I	32	13	45
3	34	F	8	RF-TC	I	16	11	43
4	27	F	7	RF-TC	I	12	15	36
5	22	M	7	ATL	I	12	4	29
6	20	F	6	RF-TC	I	9	14	34
7	34	M	6	RF-TC	I	9	14	40
8	22	F	6	RF-TC	I	9	12	18
9	30	M	7	RF-TC	I	12	8	26
10	21	F	7	RF-TC	I	18	14	23
Total	-	-	-	-	-	-	120	349

Note: RF-TC, radiofrequency thermocoagulation; ATL, anterior temporal lobectomy.

**Table 4:** Parameters values in this study.

Random Forest	Num of Estimators	Max Depth	Learning Rate	Class Weight
	100	3	0.1	Balanced
DNN classifier	**Layer**	**In**	**Out**	**Dropout**
	Fully Connected (FC)1	Feature num	128	0.3
	FC2	128	128	-
	FC3	128	128	0.3
	FC4	128	Class num	-
	**Training Epochs**	**Batch Size**	**Optimizer**	**Learning Rate**
Trainer	20	4	NAdam	1 × 10^−4^

**Table 5:** Individual patient experimental results using the proposed method MEBM-SC.

Patient ID	Training & Valid/Testing ID	TP	TN	FP	FN	SEN [%]	SPE [%]	ACC [%]	PPV [%]	NPV [%]
Pt1	Pt2-10/Pt1	14	55	0	1	93.33	100	98.57	100	98.21
Pt2	Pt1,3-10/Pt2	13	39	6	0	100	86.67	89.66	68.42	100
Pt3	Pt1-2,4-10/Pt3	8	42	1	3	72.73	97.67	92.59	88.89	93.33
Pt4	Pt1-3,5-10/Pt4	14	33	3	1	93.33	91.67	92.16	82.35	97.06
Pt5	Pt1-4,6-10/Pt5	3	29	0	1	75	100	96.97	100	96.67
Pt6	Pt1-5,7-10/Pt6	14	27	7	0	100	79.41	85.42	66.67	100
Pt7	Pt1-6,8-10/Pt7	14	36	4	0	100	90	92.59	77.78	100
Pt8	Pt1-7,9-10/Pt8	10	15	3	2	83.33	83.33	83.33	76.92	88.24
Pt9	Pt1-8,10/Pt9	6	24	2	2	75	92.31	88.24	75	92.31
Pt10	Pt1-9/Pt10	14	19	4	0	100	82.61	89.19	77.78	100
Mean		-	-	-	-	**89.27**	**90.37**	**90.87**	**81.38**	**96.58**

**Table 6:** The ablation study demonstrates that the features in our pipeline are efficient for detecting SOZ contacts.

Multi Biomarkers Features	Spike	HFOs	Pathological HFOs	Regional+10% Based Pathological HFOs	SEN [%]	SPE [%]	ACC [%]	PPV [%]	NPV [%]
feature a	√				78.80	89.95	87.74	72.89	93.37
feature b		√			74.91	91.22	88.34	68.74	93.08
feature c	√	√			82.89	89.66	88.93	73.82	95.21
feature d	√		√		87.93	89.92	90.71	71.43	93.18
**feature e/** **MEBM-SC**	√			√	**89.27**	90.37	**90.87**	**81.38**	**96.58**

**Table 7:** The ablation study shows the effectiveness of the feature selection method and classifier with other methods.

Feature Selection Methods	SEN [%]	SPE [%]	ACC [%]	PPV [%]	NPV [%]
XGBoost	56.52	95.89	84.91	71.63	86.13
RFE-XGBoost	65.81	95.02	87.89	79.75	89.54
ShapHT++DNN	86.91	89.64	89.63	75.97	95.64
ShapHT++self-AM+DNN	88.94	86.13	88.20	72.32	96.25
**MEBM-SC** (ShapHT++AM+DNN)	89.27	90.37	90.87	81.38	96.58

**Table 8:** Cross-subject validation of MEBM-SC and state-of-the-art methods.

Authors	Methods	SEN [%]	SPE [%]	ACC [%]	PPV [%]	NPV [%]
Mruphy et al. [35]	HFOs-based contact ranking index	-	-	70	-	-
Su Liu et al. [16]	HFOs-based clinical analyses	81	82.2	-	-	-
Zelin Fang et al. [36]	HFOs-Based Quantity Deviation and Semi-Maximum	60.0	96.3	-	-	-
Jian Li et al. [14]	“fingerprint”, fast activity and SVM	13.59	99.64	76.38	89.36	75.84
Zweiphenning et al. [18]	physiological ripples correction and thresholds	27	97.1	77.3	80.6	78.2
**Proposed**	DL-based multi biomarkers detection and contact classification	**89.27**	90.37	**90.87**	81.38	**96.58**

## Data Availability

The clinical dataset supporting the conclusions of this study is accessible from the authors upon reasonable request.

## Supplementary Materials

The following supporting information can be downloaded at: https://www.mdpi.com/article/10.3390/bioengineering9120769/s1, Table S1: High-frequency band(80-250Hz) features; Figure S1: The structure of 1D-CNN; Table S2: The structure of SEEG-Net; Table S2: Feature extraction of unfixed-length signal segment.

## Abbreviations

CNN = convolution neural network; DL = Deep Learning; DRE = drug-resistant epilepsy; HFO = high-frequency oscillations; ML = Machine Learning; MRI = magnetic resonance imaging; SF = Seizure free; NSF = None seizure free; SEEG = stereoelectroencephalography.
