# Supplementary material for: Automatic Localization of Seizure Onset Zone Based on Multi-Epileptogenic Biomarkers Analysis of Single-Contact from Interictal SEEG
Source: Bioengineering (Basel). 2022 Dec 5;9(12):769. doi: 10.3390/bioengineering9120769 (PMC9774098; doi:10.3390/bioengineering9120769)
Supplement: Supplementary file 1 [file bioengineering-09-00769-s001.zip › bioengineering-2044061-supplementary.pdf]

Title: Automatic Localization of Seizure Onset Zone Based on Multi-Epileptogenic Biomarkers Analysis of Single-Contact from Interictal SEEG

Supplementary Materials

Table S1. High-frequency band(80-250Hz) features.

**High DWT**	
Kraskov entropy	Energy
Renyi entropy	SVD entropy
Permutation entropy	PFD
Sample entropy	KFD
Shannon entropy	HFD

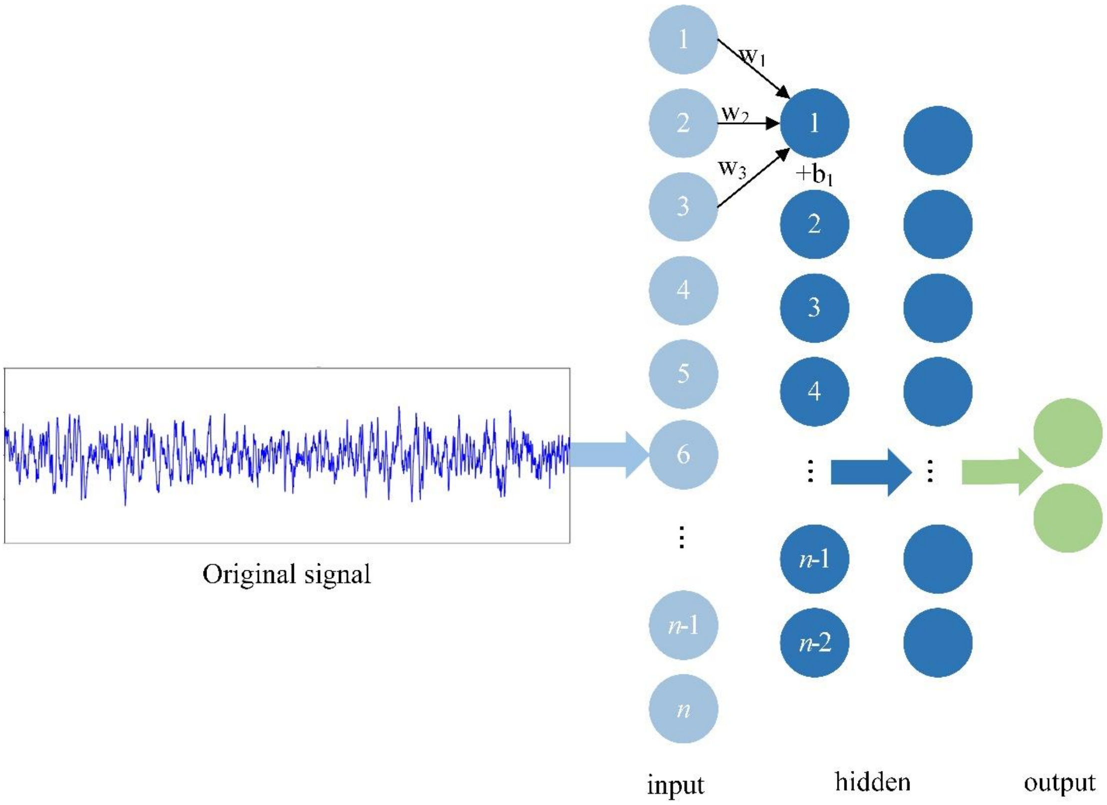

Figure S1. The structure of 1D-CNN.

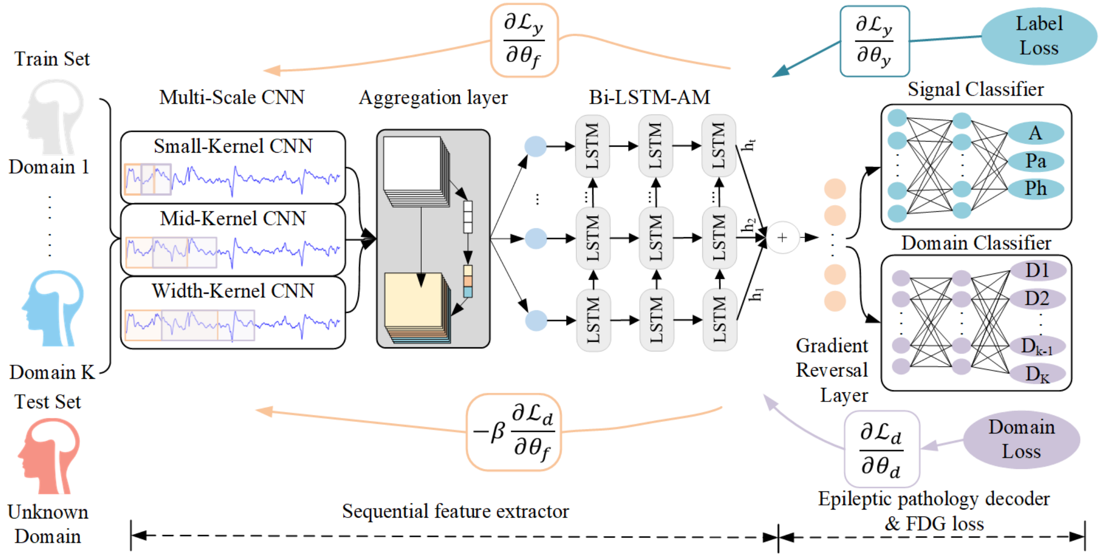

Figure S2. The structure of SEEG-Net.

Table S2. Feature extraction of unfixed-length signal segment.

Category	Num	Name	Formula
**Time domain**	1	Energy	$E = \sum_{i=1}^{N} {x_i}^2$
	2	Time	t_i
	3	Maximum	$max = max\,(fft(x_i))$
	4	Mean	$mean = \frac{1}{N}\sum_{i=1}^{N} fft(x_i)$
	5	Minimum	$min = min\,(fft(x_i))$
	6	Peak frequency	$peak = max\big(fft(x_i)\big) - min\,(fft(x_i))$
	7	Power	$P = \sum_{i=1}^{N} fft({x_i}^2)$
**FT domain**	8	Power spectral density	$s_{xx}(\omega) = \lim_{T\to\infty} E\left[\left\|\hat{X}(\omega)\right\|^2\right]$
	9	Spectral entropy	$SE = \frac{-\sum_{i=1}^{N} P_i \cdot \log P_i}{\log(length(P_i))}, P = \frac{x_i}{\sum_{i=1}^{N} x_i}$
	10	Spectrum	$spec\_max = max\,(\|fft(x_i)\|)$
	11	Variance	$\sigma^2{}_f = \frac{1}{N}\sum_{i=1}^{N}(fft(x_i) - \mu)^2$
			$L_i = \sum_{i=1}^{N}(x_i - \mu)$
**DWT**	12	Kraskov entropy	$\widehat{KE}(X) = -\varphi(k) + \varphi(N) + \log(V_d)$

**domain**			$+\cdots\frac{d}{N}\sum_{i=1}^{N}\log\big(2\delta(x_i,k)\big),$ $V_d=\pi^{d/2}/\Gamma(1+d/2)/2^d\,,k=4$
	13	Renyi entropy	$RE_\alpha(X)=\frac{1}{1-\alpha}\log\left(\sum_{i=1}^{N}p_i^\alpha\right),\alpha=2$
	14	Permutation entropy	$PE_D=-\frac{1}{\log_2 D!}\sum_{i=0}^{D!}p_i\log_2 p_i\,,D=3$
	15	Sample entropy	$SaE=-\ln\left[\frac{(N-k+1)^{-1}\sum_{i=1}^{N-k+1}A_i^k(r)}{(N-m+1)^{-1}\sum_{i=1}^{N-m+1}B_i^m(r)}\right],$ $m=2,r=0.2\times\lvert\sigma\rvert,k=m+1$
	16	Shannon entropy	$ShE(X)=\lim_{\alpha\to 1}RE_\alpha(X)=-\sum_{i=1}^{N}p_i\log p_i$
	17	Energy	$E=\sum_{i=1}^{N}{x_i}^2$
	18	SVD Entropy	$Svd=-\frac{1}{\log(N)}\sum_{j=1}^{N}\left(\frac{s_j^2}{\sum_k s_k^2}\right)\log\left(\frac{s_j^2}{\sum_k s_k^2}\right)$
	19	PFD	$P_D=\frac{\log n}{\log n+\log\left(\frac{n}{n+0.4N}\right)}$
	20	KFD	$K_D=\frac{\log n}{\log\left(\frac{d}{L}\right)+\log n}$
	21	HFD	$H_D=\ln\left(\sum_{m=1}^{k}L_m(k)\right)$
